# Methylation at CpG sites related to growth differentiation factor-15 was not prospectively associated with cardiovascular death in discordant monozygotic twins

**DOI:** 10.1038/s41598-022-08369-9

**Published:** 2022-03-15

**Authors:** Spencer Shawn Moore, Pallavi Mukherji, Ming Leung, Catherine E. Vrentas, Melsa M. Mwanja, Jun Dai

**Affiliations:** 1 Doctoral Program of Osteopathic Medicine, College of Osteopathic Medicine, Des Moines University, Des Moines, IA 50312 USA; 2 Emergency Medicine Residency Program, Loyola University Medical Center, Maywood, IL 60153 USA; 3 Institute for Personalized Medicine, Penn State College of Medicine, Hershey, PA 17033 USA; 4 Master Program of Public Health, Des Moines University, Des Moines, IA 50312 USA; 5 Oregon State Department of Human Services, Salem, OR 97303 USA; 6 Department of Public Health, College of Health Sciences, Des Moines University, 3200 Grand Avenue, Des Moines, IA 50312 USA

**Keywords:** Biomarkers, Cardiology, Risk factors

## Abstract

Myocardial infarction patients had decreased methylation at four growth differentiating factor-15 (GDF-15) related CpG sites (cg13033858, cg16936953, cg17150809, and cg18608055). These sites had not been studied for their association with cardiovascular disease (CVD) deaths. Thus, we aimed to assess the associations independent of genes, shared environment, and traditional CVD risk factors. Nineteen white, male, monozygotic twin pairs discordant for CVD deaths were included from the National Heart, Lung and Blood Institute Twin Study (NHLBI) initiated in 1969. Data on vital status was collected through December 31, 2014. Methylation of buffy coat DNA at exam 3 (1986–87) was measured using the Illumina HumanMethylation450 BeadChip. Principal component analysis was used to generate a score representing blood leukocyte composition and baseline CVD risk factors and predominated with natural killer cells, CD4+ T cells, and Framingham risk score. Conditional logistic regression demonstrated that methylation at the four CpG sites was not associated with CVD deaths before (all p > 0.05, bootstrapped p > 0.05) and after adjustment for the score (all p > 0.05). Joint influences of cg16936953 and the score were statistically significant (p < 0.05). In conclusion, joint influences of methylation at the site cg16936953 and the score are prospectively associated with CVD deaths independent of germline and common environment.

**ClinicalTrials.gov Identifier for NHLBI Twin Study:** NCT00005124.

## Introduction

Cardiovascular disease (CVD) is the leading cause of death worldwide, with most associated deaths resulting from atherosclerotic CVD such as heart attacks or heart failure in the U.S.^1^. Increasing our understanding of the contributions of gene-environment interactions to the risk of death from CVD remains of high importance to the treatment and prevention of this disease. Epigenetic changes constitute molecular modifications to the genome beyond changes to the gene sequence itself^2^. Therefore, epigenetics can inform our understanding of these interactions. For example, epigenetic changes can provide a mechanism by which genetics, influenced by the environment, contributes to disease. Additionally, epigenetic changes can serve as a biomarker of gene-environment interactions^3^.

Epigenetic changes to the genome are stable across mitotic divisions^4^. They involve chemical modifications to DNA or associated proteins, such as the methylation of 5-cytosine in CpG (cytosine-phosphate-guanine) DNA dinucleotide pairs in the genome via the transfer of a methyl group to the pyrimidine ring of the cytosine^4^. Methylation of promoter sequences is associated with repression of expression of the associated gene(s) and establishment of longer-term gene silencing^5^. While most variation in DNA methylation patterns between individuals is due to environmental factors, genetic influences on DNA methylation are high at certain genomic sites, especially those exhibiting high levels of variability in methylation^6^.

In a genome-wide methylation association study, after adjusting for white blood cell composition, methylation levels at four CpG sites (cg17150809, cg13033858, cg16936953, and cg18608055) were associated with circulating GDF-15 levels in discovery and validation populations and were potentially associated with prior myocardial infarction history^7^. Differential DNA methylation has been associated with expression levels of growth differentiation factor-15 (GDF-15), a biomarker for CVD risk^7^. GDF-15 is a member of the transforming growth factor-β family involved in regulating inflammatory processes^8^. A recent meta-analysis of 31 prospective studies demonstrated that higher levels of circulating GDF-15 were associated with a higher risk of cardiovascular mortality [hazard ratio (HR) 2.11; 95% confidence interval (CI) 1.57–2.66 per log-unit ng/L increment] among 53,706 individuals who had an average age of 60–75 years^9^.

Although the associations of methylation at the four GDF-15 related CpG sites with the prior myocardial infarction history became statistically insignificant after multiple-testing adjustment^7^, the sites are of interest for further studies because potential residual confounding, such as genetic factors, could mask the association. Epigenetic association studies for CVD in the general population are potentially confounded by genetic factors, as genetic variation impacts human DNA methylation patterns^10^ and CVD^11^. Prior studies on the influence of human DNA methylation at these GDF-15 related CpG sites on CVD hard outcomes are rare. Importantly, previous few studies demonstrate inconsistent findings of methylation at the GDF-15 related CpG sites in relation to CVD risk factors among humans, suggesting potential genetic confounding. For example, a prior study of blood levels of C-reactive protein (CRP) as a biomarker for chronic low-grade inflammation towards CVD reported inconsistent directions of association of the site cg16936953 but not cg18608055 with serum CRP between individuals of European ancestry and those of African Americans^12^. Inconsistent associations were also reported between methylation at the CpG site cg18608055 and obesity as a CVD risk factor^13,14^. A negative association was found among middle-aged African Americans from the Atherosclerosis Risk in Communities (ARIC) study^13^ and among young Norwegian women^14^; however, the negative association was unable to be replicated in Finnish monozygotic twins discordant for body mass index^15^, implying that the observed association might be due to potential confounding from germline and shared environment.

Discordant monozygotic (MZ) twin pair studies, in which one member of the twin pair (i.e., one co-twin) is affected by a disease and the other is not, provide an optimal, natural experiment to control for genetic confounding because of the identical genomic sequence between co-twins of a monozygotic twin pair^16^. In addition, twin studies also control for age and shared environmental factors^17^, such as the prenatal environment, and thus can provide significant power to detect associations between epigenetic patterns and complex diseases^18^.

This study used a discordant MZ twin design to include MZ twins discordant for CVD death from the 41-year follow-up National Heart, Lung, and Blood Institute (NHLBI) Twin Study. We aimed to assess whether methylation at the four genomic CpG sites linked to circulating GDF-15 levels was prospectively associated with CVD death risk independent of germline, shared environmental factors, blood leukocyte composition, and known traditional CVD risk factors.

## Results

### Characteristics of study participants

All 19 control co-twins outlived their case co-twin brothers in this study, of which 18 control co-twins died before the date of the end of follow up (i.e., December 31, 2014) and one control co-twin was still alive on December 31, 2014. The diseased twins died significantly earlier than their co-twin brothers (74.7 versus 83.7 years old at death on average) with the within-pair differences (i.e., pair-wise differences) in age at death or the last follow-up date (years) ranging from 3.5 to 23.8 years [mean ± SD 10.3 ± 5.7 years; median (IQR) 10.0 (9.3) years] for control twins relative to their diseased identical twin brothers (Table 1 and Supplementary Fig. S1). Diseased twins had significantly higher systolic blood pressure (p = 0.03) and lower levels of low-density lipoprotein cholesterol (p = 0.008) at baseline than their control twin brothers.

**Table 1:** Characteristics of monozygotic twin pairs discordant for cardiovascular death *CVD-dMZ* monozygotic twin pairs discordant for CVD death, *HDL-C* high-density lipoprotein cholesterol, *LDL-C* low-density lipoprotein cholesterol. Variables (%) are dichotomous except smoking status. Obesity is defined as body mass index at or greater than 30. Data at baseline are presented as mean (SD) unless otherwise specified. Raw values for continuous variables are presented. All probability values are corrected for pair clustering. Mixed models were used for continuous variables, conditional logistic models for dichotomous variables, and repeated proportional odds model with generalized estimating equation for the 3-level ordinal smoking variable. The conversion of cholesterol from mg/dL to mmol/L is divided by 38.67. The conversion of glucose from mg/dL to mmol/L is divided by 18.02.

Characteristics	CVD-dMZ (n = 19 pairs)			p value
	Total	Case twins	Control twins	
Discordant pairs, n	19	19	19	–
Age at baseline (year)	50.4 (2.5)	50.4 (2.5)	50.4 (2.5)	1.00
Age at death (year)	79.0 (7.9)	74.3 (5.7)	83.7 (7.1)	< 0.0001
**Cigarette smoking, n (%)**				0.85
Never smokers	15 (39)	7 (37)	8 (42)	
Former smokers	6 (16)	3 (16)	3 (16)	
Current smokers	17 (45)	9 (47)	8 (42)	
Education, (year)	13.9 (2.6)	14.2 (3.1)	13.6 (2.2)	0.36
Body mass index (kg/m^2^)	27.3 (3.7)	27.4 (3.6)	27.2 (3.8)	0.58
Obesity, n (%)	5 (13)	1 (5.3)	4 (21)	0.25
Systolic blood pressure (mmHg)	133 (17)	138 (18)	128 (13)	0.03
Diastolic blood pressure (mmHg)	84 (10)	87 (10)	82 (10)	0.058
**Plasma biochemistry**				
HDL-C/triglyceride	0.38 (0.22)	0.36 (0.22)	0.39 (0.22)	0.58
LDL-C (mg/dL)	147 (43)	138 (43)	155 (42)	0.008
Postload glucose (mg/dL)	156 (43)	160 (53)	153 (30)	0.41
Framingham risk score, unit	6.0 (1.8)	5.8 (1.9)	6.2 (1.7)	0.42
Diabetes, n (%)	1 (2.6)	1 (5.3)	0 (0)	1.00
Use of antihypertensives, n (%)	7 (18)	4 (21)	3 (16)	1.00
Interval between exam 3 and death/end of follow-up (year)	13.0 (7.13)	8.43 (5.23)	17.5 (5.82)	< 0.0001
**Peripheral blood leukocyte composition estimated from methylation data at exam 3, (%)**				
CD8+ T-cells	2.6 (3.2)	3.0 (3.7)	2.2 (2.7)	0.44
CD4+ T-cells	13.9 (5.7)	13.0 (5.4)	14.8 (5.9)	0.11
Natural killer cells	5.6 (4.1)	5.7 (4.6)	5.6 (3.8)	0.87
B-cells	4.1 (1.8)	4.1 (2.1)	4.1 (1.6)	0.94
Monocytes	7.1 (3.2)	7.0 (2.9)	7.1 (3.4)	0.91
Granulocytes	68.3 (7.9)	68.7 (8.8)	67.9 (7.0)	0.69

### Associations of methylation at the four CpG sites with risk for CVD death

Table 2 shows that methylation levels at the 4 CpG sites, cg13033858, cg16936953, cg17150809, and cg18068055, were not statistically significantly associated with the risk of death from total CVD (all p > 0.05) before and after controlling for the principal component score as a surrogate score with wide 95% CIs. However, the joint influence of cg16936953 and the score was statistically significantly associated with CVD death (p < 0.05), and the adjusted hazard ratio (HR) was marginally statistically significant for cg16936953 [HR 4.38, 95% confidence intervals (CI) 0.79–24.3, p = 0.09] and the score (p = 0.06), respectively. The information gain from the joint influence of CpG sites and the surrogate score above 10% was 13% and 10% for cg16936953 and cg17150809, respectively, suggesting the biological, clinical, or scientific importance of the joint influences associated with CVD death risk (Table 2). The varimax-rotated weight for the score was − 0.78, 0.67, 0.65, − 0.40, − 0.25, 0.05, − 0.097, and 0.06 for natural killer cells, CD4+ T cells, Framingham risk score, CD8+ T cells, body mass index, B cells, monocytes, and years of education, respectively, suggesting the biological and clinical importance of natural killer cells, CD4+ T cells, and Framingham risk score.

**Table 2:** Hazard ratio per 10% unit increase in methylation beta value derived from the regression coefficient from a conditional logistic model among monozygotic twins discordant for total cardiovascular death. *CI* confidence interval. Adjusted hazard ratios were obtained after control for a principal component score representing age, years of education, smoking status, body mass index, systolic blood pressure, low-density lipoprotein cholesterol, high-density lipoprotein cholesterol, use of antihypertensives, presence of diabetes, and leukocyte subtypes.

	Hazard ratio (95% CI)	p value	Information gain
**cg13033858**			
Crude	0.64 (0.18, 2.22)	0.48	1.4%
Adjusted	1.23 (0.25, 6.04)	0.80	5.3%
**cg16936953**			
Crude	2.14 (0.64, 7.22)	0.22	4.7%
Adjusted*	4.38 (0.79, 24.3)	0.09	12.8%
**cg17150809**			
Crude	1.60 (0.35, 7.21)	0.54	1.0%
Adjusted	3.48 (0.46, 26.5)	0.23	10.3%
**cg18608055**			
Crude	1.08 (0.28, 4.07)	0.91	0.03%
Adjusted	2.28 (0.42,12.3)	0.34	9.1%
**Score**			
Crude	0.48 (0.15, 1.57)	0.22	4.6%

*p < 0.05 likelihood ratio test for the adjusted model in which the score had an adjusted p-value of 0.06.

Statistical insignificant results were found among stringently defined twin pairs discordant for CVD, primarily and stringently defined twin pairs discordant for coronary heart death (Supplementary Table S1).

### Bootstrap analysis

Methylation levels at the 4 CpG sites were not statistically significantly associated with the risk of death from total CVD (all bootstrapped p > 0.05) without controlling for the surrogate score in the bootstrap analysis of 10,000 bootstrapped samples. The bootstrapped 95% CI was (0.37–1.47), (0.82–1.81), (0.55–3.58), and (0.57–2.01) after natural adjustment for germline and shared environment for sites cg13033858, cg16936953, cg17150809, and cg18608055, respectively.

## Discussion

After natural adjustment for germline and common environment, methylation levels at the 4 CpG sites, cg13033858, cg16936953, cg17150809, and cg18068055, were not statistically significantly associated with death from total CVD before and after additional controlling for potential confounding from blood leukocyte composition and traditional known CVD risk factors in the original sample as well as after natural controlling for germline and shared environment only in 10,000 bootstrapped samples. Unadjusted bootstrapped 95% CIs indicated the precision of estimates at sites cg17150809 and cg18608055 were very poor compared to that at sites cg13033858 and cg16936953, implying the sites cg13033858 and cg16936953 might be more clinically useful, particularly the site cg16936953 with the narrowest 95% CI among the 4 CpG sites. From a clinical view, influences of 10% increment in methylation β values on the CVD survival ranged from 18% protective to 81% detrimental at cg16936953, independent of germline and common environment. In addition, in the original sample, the joint association of the CpG sites and the overall effect of blood leukocytes and traditional known CVD risk factors with CVD death risk was biologically/clinically important for cg17150809 while additionally statistically significantly important for cg16936953, independent of germline and common environment.

Previous human population studies of potential methylation associations at the four sites with hard cardiovascular outcomes are few. In addition to the previous study of the association of the four CpG sites with myocardial infarction as a hard outcome^7^, a meta-analysis showed that methylation levels of the site cg18608055 in the blood DNA were not associated with incident acute coronary syndrome among Chinese^19^. We did not find previously published research on methylation at sites cg17150809, cg13033858, or cg16936953 related to other hard CVD outcomes in humans. Therefore, our study provides unique evidence on cardiovascular death as a CVD hard outcome independent of germline, common environment, blood leukocyte composition, and traditional CVD risk factors, in particular, evidence of the potential joint influence of the CpG sites and the overall effect of blood leukocyte composition and traditional CVD risk factors on cardiovascular death.

Given the 95% confidence intervals, we explored the possible pathophysiological mechanisms linking methylation at the 4 CpG sites to CVD although the mechanisms are poorly understood. Besides the regulatory production of GDF-15 via the methylation at the four sites as a possible mechanism^7^, there are other potential mechanisms. Inflammation and biological processes like vessel remodeling are involved in atherogenesis and fibrosis underlying atherosclerotic CVD, hypertension, and heart failure^20,21^, which are the main causes of CVD deaths in the U.S.^1^. Therefore, it is speculated that methylation at the four sites is involved in atherosclerosis and fibrosis via gene-silencing of relevant genes.

The site cg13033858 is located on chromosome 12 within the gene body coding for protein phosphatase slingshot homolog-1 (SSH1) and related to the CpG shelf, 2 to 4 kb from the upstream of the CpG island. SSH1 may reduce fibrosis by suppressing angiotensin II-induced remodeling in the vasculature through regulating actin filament dynamics and SSH1 activity^22^. Methylation at the site cg13033858 would thus enhance fibrosis by downregulating SSH1 biosynthesis, leading to heart failure.

The site cg17150809 is located on chromosome 6 and upstream of the gene coding for F-Box and Leucine Rich Repeat Protein 4 (FBXL4)^7^. FBXL4 protein suppresses mitochondrial degradation through its function related to E3 ubiquitin ligase complex activity^23^. Bioinformatics analysis revealed that the FBXL4 gene in peripheral blood monocytes might contribute to atherosclerosis^24^. As reviewed by Kapnick et al.^25^, the reduction in FBXL4 protein contributed to immune dysfunction, including neutropenia, lymphopenia, and frequent infections, and reductions in natural killer cells, total CD8+ T cells, and CD8+ memory T cells. Our findings suggested the potential biological and clinical important role of natural killer cells and CD4+ T cells, to a lesser extent CD8+ T cells, along with this site cg17150809 in cardiovascular death contributing to the pathophysiological process.

Sites cg16936953 and cg18608055 appear to have bi-directional roles in CVD. First, the site cg16936953 was located on chromosome 17 within the Vacuole membrane protein 1 (*VMP1*)/Micro-RNA 21 (*MIR21*)/promoter to regulate miRNA-21 biogenesis. MiRNA-21 plays crucial but controversial roles in CVD^26^. Its controversial cardiovascular functions might depend on specific cell subtypes and the pathophysiological stages in cardiovascular impairment^26^. MiRNA-21 could protect cardiac function by preventing excessive inflammation and cardiac dysfunction after myocardial infarction through targeting KBTBD7^27^. Thus, downregulation of miRNA-21 biogenesis by methylation at the site cg16936953 could be detrimental to cardiac function recovery. On the other hand, miRNA-21 could promote the progress of cardiac hypertrophy to heart failure by mediating cardiac fibrosis. Therefore, downregulation of miRNA-21 biogenesis by methylation at this site could prevent the hypertrophic heart from heart failure^26^. Our findings suggested the potential statistical and clinical importance of the surrogate score, particularly natural killer cells, CD4+ T cells, and Framingham risk score, in their joint effect with the site cg16936953 on the cardiovascular death contributing to the pathophysiological process.

Second, the CpG site cg18608055 is located on chromosome 19 within the body of the gene “Strawberry notch homologue 2 (SBNO2).” Evidence from laboratory experiments suggested that SBNO2 was involved in both inflammation and anti-inflammation^28,29^. SBNO2 was an acute inflammatory response gene upregulated by IL-6^28^, while SBNO2 protein had strong repressive activity for NF-κB and was a component of the pathway regulating the downstream anti-inflammatory effects of interleukin-10^29^.

Overall, the pathophysiological mechanisms are putative as methylation at the four sites could regulate the biogenesis of factors in the regulatory network contributing to atherosclerosis and fibrosis. Thus, more laboratory research is needed to elucidate pathophysiological mechanisms linking methylation at these sites to CVD disease.

There are limitations to this study. Our discordant MZ twin sample size seems small. As described in our previous study^30^, the twin birth was less than 1.5 in 100 births during 1917 to 1927 when twins in the NHLBI Twin Study were born^31–33^. Further considering male MZ twin live birth rate^33^ and live births^34^ during the same period in the US, we estimated that the 254 MZ pairs initially enrolled in the NHLBI Twin Study were comparable to 110,535 male singletons^30^. Since the Framingham Heart Study of cardiovascular disease enrolled 2344 men in its initial cohort^35^, our sample size was large when we took twinning birth rate and male monozygotic twin live birth rate into consideration^30^. The extremely long follow-up in the NHLBI Twin Study provided a unique opportunity to prospectively study cardiovascular death in the large discordant twin cohort. However, we cannot exclude the potential statistical insignificance because of insufficient statistical power resulting from the small sample size. Our results could be used to estimate the sample size for future large-scale studies. Second, we used the first principal analysis component as a surrogate for the overall effect of blood leukocytes and known CVD risk factors to minimize overfitting. The first component explained less than 70% of the variation in blood leukocytes and known CVD risk factors. Although we could not exclude residual confounding, the most parsimonious model was the first principal component adjusted model for the four CpG sites (Supplementary Table S2). We did not measure messenger RNA (mRNA) to evaluate GDF-15 expression since the technique to preserve mRNA was not used in the mid-1980s when biospecimens were collected in the NHLBI Twin Study. We were unable to evaluate the between-pair effects because of the use of the discordant twin pair design and the resultant conditional logistic regression analysis.

Our study has several advantages. The use of MZ discordant twin pairs allowed for an assessment of associations in the absence of genetic confounding, as MZ twin pairs are the same germline. In addition to adjustment for age-cohort-period influences in our discordant twin design, we controlled for traditional known CVD risk factors to a certain degree, including years of education representing socioeconomic status, body mass index, age, systolic blood pressure, low-density lipoprotein cholesterol, high-density lipoprotein cholesterol, current cigarette smoking status, use of antihypertensives, and the presence of type 2 diabetes. We provided the unadjusted 95% CIs from 10,000 bootstrap resamples as an alternative to minimize potentially biased results because of the relatively small sample size^36^. To further reduce the bias, a larger sample of discordant identical twin pairs would be needed. Our extended longitudinal design demonstrated that methylation temporally occurred before cardiovascular death. This temporal order was critical to explain the causal role of methylation at the studied 4 CpG sites in cardiovascular death.

In conclusion, the joint influences of methylation at cg16936953 and the overall effect of blood leukocyte composition (particularly, natural killer cells and CD4+ T cells) and known CVD risk factors (particularly, Framingham risk score) are prospectively associated with the risk of death from total CVD, independent of genetic and shared environmental factors.

## Methods

### Study population

The NHLBI Twin Study was a longitudinal cohort study designed to study genetic and environmental causes of cardiovascular disease in the United States^37^. The original cohort was initiated in 1969 and included 514 middle-aged white male veteran twin pairs (1028 men, 254 monozygotic and 260 dizygotic twin pairs) from the National Academy of Sciences-National Research Council Veteran Twin registry who lived within 200 miles of five research centers: Framingham, Massachusetts; San Francisco, California; Davis, California; Los Angeles, California; and Indianapolis, Indiana^38^. Members of the cohort were born between 1917 and 1927 and were 42–55 years of age at the baseline examination (Exam 1, 1969–1973)^17^. The baseline and subsequent examinations were conducted using the reputable Framingham Heart Study protocol to ensure uniform physical examination by experienced cardiovascular epidemiologists^17^. Five active follow-up studies were performed during 1981–1982, 1986–1987, 1995–1997, 1999–2001, and 2001–2003^39–41^. Both biochemical and questionnaire data were collected at baseline and exams 2 and 3. The last three examinations (exams 4–6) collected structural brain MRI and questionnaire data^41^. A total of 792^38^, 622^38^, 595, 438, and 174 twins participated in exams 2–6, respectively. Zygosity was identified in the 1960s using eight red blood cell antigen groups and in the 1980s using the variable number of tandem repeat DNA markers^42^.

This reported study used a discordant monozygotic twin pair design that included identical twin pairs discordant for CVD death. As described in our previous study^30^, this approach was a specific type of the 1:1 individually matched, nested case–control design, in which one co-twin within a twin pair was the case and his co-twin brother was the control. Thus, this prospective design could be called a nested case-co-twin-control design. In the nested case–control design^43^, a control had not developed the disease by the time of disease occurrence in the case (index date) and might later become a case^43^. The advantage of this definition was “to make inference under a proportional hazards model from the conditional logistic approach”^44^. Thus, this design could provide unbiased relative risk estimates^45^. We used the following definitions to identify twin pairs discordant for CVD. The primary definition of a twin pair discordant for total CVD death was one where a co-twin died from CVD (case co-twin) and his co-twin brother who did not die from CVD on the index date^46^ or died from CVD at least 1 year later (control co-twin). A more stringent definition was one where a co-twin died from CVD (case) and his co-twin brother did not die from CVD by the end of the follow-up (control). Inclusion criteria included: (1) available buffy coat DNA ≥ 400 µg on the DNA inventory of Veteran Twin Samples after exam 5 for each co-twin of a twin pair, (2) available data on vital status, dates of death, and causes of death through December 31, 2010; and (3) methylation and hydroxymethylation were measured. We performed zygosity-specific stratified random sampling by twins discordant for coronary heart death and non-coronary heart CVD death. Vital data were updated in 2014. The primarily-defined discordant twin pairs using 2010 and 2014 data were identical. Thus, we finally included 19 monozygotic twin pairs (CVD-dMZ) discordant for CVD death. This study was approved by the Institutional Review Board (IRB) of Vanderbilt University as a non-human subject study due to the use of de-identified information (IRB #141163, July 25, 2014), and informed consent was also waived by this IRB. In addition, the approval from the IRB of Des Moines University was waived due to the prior approval at Vanderbilt University. All methods were performed in accordance with the relevant guidelines and regulations.

### DNA sample collection

As previously published, buffy coat DNA samples were collected at exam 3 (1986–1987) in the NHLBI Twin Study^30,41^. Whole blood was drawn from the antecubital vein into EDTA tubes after an overnight fast and immediately placed on ice. Buffy coats were obtained and used to extract DNA. Spectral analysis was used to determine the quantity and quality of the extracted DNA. DNA samples were stored at − 70 °C. All samples were labeled by the study number only.

### Genome-wide methylation (5mC) measures

Genome-wide methylation was measured with the Illumina Infinium HumanMethylation450 (450 K) BeadChip array following the established Illumina protocol. Co-twin samples were processed in the same analytical run without knowing disease status to minimize measurement error. For quality control, beta values with associated detection p-values greater than 0.01 were set to missing. All samples passed quality control (missing percentage < 1.5%). CpGs with greater than 10% missing data were removed (452 CpGs removed)^47,48^.

### Other known cardiovascular risk factors at baseline

All major cardiovascular risk factors were recorded on a questionnaire through in-person interviewing and physical examination at baseline (exam 1, 1969–1973)^17^. Participants were assessed on demographic, lifestyle, familial, socioeconomic, biochemical, and clinical factors. Age, years of education, marital status, and smoking status were recorded, and physical exam measurements included height, weight, diastolic and systolic blood pressures, and a 12-lead electrocardiogram. Lab measurements included 9-h fasting glucose levels and a glucose load tolerance test. Low-density lipoprotein cholesterol levels were calculated using the Friedewald equation^49^. Physicians assessed the history of heart and other cardiovascular diseases and cardiovascular events and procedures.

### Follow-up and assessment of endpoints

Vital status and the cause and date of death were ascertained through medical records in the five active follow-up examinations^41^ and later using death certificates or the National Death Index through December 31, 2014. As previously described^50,51^, physicians assigned corresponding International Classification of Diseases, Ninth Revision codes (ICD-9) for morbidity outcomes. Death certificates or the National Death Index coded to the ninth revision codes were obtained for decedents. The endpoint was death from all cardiovascular diseases (390–398, 402, 404, 410–438). Subjects were considered lost to follow-up if a death certificate or coding from the National Death Index could not be traced. The follow-up was terminated at the date of death, end of follow-up, or loss to follow-up, whichever occurred first.

### Statistical analysis

The post-normalized beta values were calculated using the 450 K array methylation data following the published method^47^. Briefly, data was batch normalized using the Combat function in R using a set of 12 samples on a single array as a batch. To carry out the normalization, parallel processing was used on random subsets of 20,000 CpGs. Probes of different chemistries (Infinium I or Infinium II) were normalized separately. After normalization, a chemistry correction was applied. This correction was based on applying a second-order polynomial fit using CpGs of different chemistries within 50 bp of each other (correlations > 0.99 for pairs of CpGs within this distance). The missing value for a co-twin was replaced with his co-twin brother’s value. Only one participant had missing values for lipid profile and Framingham risk score, while another participant had a missing value for the beta value at cg17150809.

Principal component analysis with varimax rotation was performed on blood leukocytes and known CVD risk factors. Five of the six types of blood leukocytes (i.e., CD4+ T-cells, CD8+ T-cells, natural killer (NK) cells, B-cells, monocytes, and granulocytes), except for granulocytes, were selected a priori^52^ as the sum of the composition of six types of blood leukocyte composition was equal to 100%. CVD risk factors included years of education (continuous), body mass index (continuous) and the calculated Framingham risk score, which incorporated factors including age, systolic blood pressure (continuous), total cholesterol (continuous), high-density lipoprotein cholesterol (continuous), use of blood pressure medication (yes/no), cigarette smoker (yes/no), and presence of diabetes (yes/no)^53^. To avoid overfitting, we used the first principal component score as the surrogate measure to reflect the potential confounding from the leukocyte and CVD risk factors.

We used conditional logistic regression to calculate hazard ratios for evaluating the association of the continuous methylation level in β values with CVD death. We first evaluated the association without adjustment for any covariates. Then, we adjusted for the first principal component score. The information gain from adding predictors such as CpG site and the surrogate score into the model was calculated as the difference between the negative of twice the log likelihood (− 2LogL) from a model without predictors and − 2LogL from the model with the predictors (CpG in the unadjusted model or CpG and the score in the adjusted model) divided by twice the number of twin pairs, and then multiplied by 100^54^. This sample size adjusted information gain reflects the scientific and practical importance of the predictor^54^.

Lastly, a bootstrap resampling analysis was performed as previously described^36^. Bootstrapping randomly sampled individual twin pairs to create a new data set of equal size to the original dataset and had several statistical advantages. The simulated samples came from the actual data, making no assumptions about the parent population parameters. This method also allowed for bypassing the large sample size assumption needed for most other statistical analyses^55^. We generated 10,000 resampled data sets using the nonparametric bootstrap method. Then, unadjusted models were repeatedly fitted to each bootstrapped data set. The 95% confidence intervals (95% CIs) for hazard ratios were computed for each CpG site from the 10,000 bootstrap samples.

R and SAS version 9.4 statistical software was used for the analysis. Blood leukocyte composition was estimated for six blood leukocyte types from the 450 K array methylation data using the “estimateCellCounts” function in the R package “minfi”^56^. The “clogit” and “prcomp” functions were used for conditional logistic regression and principal component analysis, respectively. Bootstrap analysis was performed using the R package “bootstrap.” We also performed the sensitivity analysis among a subset of 13 stringently defined CVD-dMZ pairs and another subset of 13 MZ pairs primarily defined discordant for coronary heart death out of the primarily defined 19 CVD-dMZ pairs. Both subsets had an overlap of the 8 MZ pairs. As results were materially similar to those from primarily defined pairs, we reported the results from the primarily-defined twin pairs discordant for CVD here and sensitivity analysis results in the supplementary file. The statistical significance level was set at the alpha level of 0.05.

## Supplementary Information

Supplementary Information.

## Abbreviations

NHLBI: National Heart, Lung, Blood Institute
CVD: Cardiovascular disease
MZ: Monozygotic twins
GDF-15: Growth differentiating factor-15
HR: Hazard ratio
CI: Confidence interval

## References

[1] Virani SS (2021). Heart disease and stroke statistics-2021 update: A report from the American Heart Association. Circulation.

[2] Grønbaek K, Hother C, Jones PA (2007). Epigenetic changes in cancer. APMIS.

[3] Ladd-Acosta C, Fallin MD (2016). The role of epigenetics in genetic and environmental epidemiology. Epigenomics.

[4] Almouzni G, Cedar H (2016). Maintenance of epigenetic information. Cold Spring Harb. Perspect. Biol..

[5] Suzuki MM, Bird A (2008). DNA methylation landscapes: Provocative insights from epigenomics. Nat. Rev. Genet..

[6] Hannon E (2018). Leveraging DNA-methylation quantitative-trait loci to characterize the relationship between methylomic variation, gene expression, and complex traits. Am. J. Hum. Genet..

[7] Ek WE (2016). Genome-wide DNA methylation study identifies genes associated with the cardiovascular biomarker GDF-15. Hum. Mol. Genet..

[8] Bootcov MR (1997). MIC-1, a novel macrophage inhibitory cytokine, is a divergent member of the TGF-beta superfamily. Proc. Natl. Acad. Sci. U. S. A..

[9] Xie S, Lu L, Liu L (2019). Growth differentiation factor-15 and the risk of cardiovascular diseases and all-cause mortality: A meta-analysis of prospective studies. Clin. Cardiol..

[10] Gaunt TR (2016). Systematic identification of genetic influences on methylation across the human life course. Genome Biol..

[11] Kathiresan S, Srivastava D (2012). Genetics of human cardiovascular disease. Cell.

[12] Ligthart S (2016). DNA methylation signatures of chronic low-grade inflammation are associated with complex diseases. Genome Biol..

[13] Wang X (2018). An epigenome-wide study of obesity in African American youth and young adults: Novel findings, replication in neutrophils, and relationship with gene expression. Clin. Epigenetics.

[14] Kvaløy K, Page CM, Holmen TL (2018). Epigenome-wide methylation differences in a group of lean and obese women—A HUNT Study. Sci. Rep..

[15] Ollikainen M (2015). Genome-wide blood DNA methylation alterations at regulatory elements and heterochromatic regions in monozygotic twins discordant for obesity and liver fat. Clin. Epigenetics.

[16] Castillo-Fernandez JE, Spector TD, Bell JT (2014). Epigenetics of discordant monozygotic twins: Implications for disease. Genome Med..

[17] Dai J, Krasnow R, Liu L, Sawada S, Reed T (2013). The association between postload plasma glucose levels and 38-year mortality risk of coronary heart disease: The prospective NHLBI Twin Study. PLoS One.

[18] Bell JT, Spector TD (2011). A twin approach to unraveling epigenetics. Trends Genet..

[19] Long P (2021). Profile of copper-associated DNA methylation and its association with incident acute coronary syndrome. Clin. Epigenetics.

[20] Lyle AN, Taylor WR (2019). The pathophysiological basis of vascular disease. Lab. Investig..

[21] Xiao L, Harrison DG (2020). Inflammation in hypertension. Can. J. Cardiol..

[22] Williams HC (2019). The cofilin phosphatase slingshot homolog 1 restrains angiotensin II-induced vascular hypertrophy and fibrosis in vivo. Lab. Investig..

[23] Alsina D (2020). FBXL4 deficiency increases mitochondrial removal by autophagy. EMBO Mol. Med..

[24] Zhang YM, Meng LB, Yu SJ, Ma DX (2020). Identification of potential crucial genes in monocytes for atherosclerosis using bioinformatics analysis. J. Int. Med. Res..

[25] Kapnick SM, Pacheco SE, McGuire PJ (2018). The emerging role of immune dysfunction in mitochondrial diseases as a paradigm for understanding immunometabolism. Metabolism.

[26] Dai B (2020). The cell type-specific functions of miR-21 in cardiovascular diseases. Front. Genet..

[27] Yang L (2018). MicroRNA-21 prevents excessive inflammation and cardiac dysfunction after myocardial infarction through targeting KBTBD7. Cell Death Dis..

[28] Grill M (2015). Strawberry notch homolog 2 is a novel inflammatory response factor predominantly but not exclusively expressed by astrocytes in the central nervous system. Glia.

[29] El Kasmi KC (2007). Cutting edge: A transcriptional repressor and corepressor induced by the STAT3-regulated anti-inflammatory signaling pathway. J. Immunol..

[30] Dai J (2021). Whole-genome differentially hydroxymethylated DNA regions among twins discordant for cardiovascular death. Genes (Basel).

[31] Miura T, Kawana H, Nonaka K (1987). Twinning in New England in the 17th-19th centuries. Acta Genet. Med. Gemellol. (Roma).

[32] Fellman J, Eriksson AW (2005). The convergence of the regional twinning rates in Sweden, 1751–1960. Twin Res. Hum. Genet..

[33] Strandskov HH, Edelen EW (1946). Monozygotic and dizygotic twin birth frequencies in the total, the "white" and the "colored" U.S. populations. Genetics.

[34] Live Births and Birth Rates by Year. Infoplease© 2000–2012 Pearson Education. (2007). http://www.infoplease.com/ipa/A0005067.html (Accessed 23 Dec 2012).

[35] Mahmood SS, Levy D, Vasan RS, Wang TJ (2014). The Framingham Heart Study and the epidemiology of cardiovascular disease: A historical perspective. Lancet.

[36] Dai J (2009). Beneficial effects of designed dietary fatty acid compositions on lipids in triacylglycerol-rich lipoproteins among Chinese patients with type 2 diabetes mellitus. Metabolism.

[37] Feinleib M (1977). The NHLBI twin study of cardiovascular disease risk factors: Methodology and summary of results. Am. J. Epidemiol..

[38] Reed T, Carmelli D, Christian JC, Selby JV, Fabsitz RR (1993). The NHLBI male veteran twin study data. Genet. Epidemiol..

[39] Krishnan E, Lessov-Schlaggar CN, Krasnow RE, Swan GE (2012). Nature versus nurture in gout: A twin study. Am. J. Med..

[40] Gatz M (2019). The NAS-NRC twin registry and duke twins study of memory in aging: An update. Twin Res. Hum. Genet..

[41] Gatz M (2015). Cohort profile: The National Academy of Sciences-National Research Council Twin Registry (NAS-NRC Twin Registry). Int. J. Epidemiol..

[42] Reed T (2005). Verification of self-report of zygosity determined via DNA testing in a subset of the NAS-NRC twin registry 40 years later. Twin Res. Hum. Genet..

[43] Ernster VL (1994). Nested case-control studies. Prev. Med..

[44] Kim RS (2013). Analysis of nested case-control study designs: Revisiting the inverse probability weighting method. Commun. Stat. Appl. Methods.

[45] Wang MH, Shugart YY, Cole SR, Platz EA (2009). A simulation study of control sampling methods for nested case-control studies of genetic and molecular biomarkers and prostate cancer progression. Cancer Epidemiol. Biomark. Prev..

[46] Graham DJ (2005). Risk of acute myocardial infarction and sudden cardiac death in patients treated with cyclo-oxygenase 2 selective and non-selective non-steroidal anti-inflammatory drugs: Nested case-control study. Lancet.

[47] Absher DM (2013). Genome-wide DNA methylation analysis of systemic lupus erythematosus reveals persistent hypomethylation of interferon genes and compositional changes to CD4+ T-cell populations. PLoS Genet..

[48] Logue MW (2017). The correlation of methylation levels measured using Illumina 450K and EPIC BeadChips in blood samples. Epigenomics.

[49] Friedewald WT, Levy RI, Fredrickson DS (1972). Estimation of the concentration of low-density lipoprotein cholesterol in plasma, without use of the preparative ultracentrifuge. Clin. Chem..

[50] Mikulec KH (2004). Relationship of endogenous sex hormones to coronary heart disease: A twin study. J. Clin. Endocrinol. Metab..

[51] Dai J, Mukamal KJ, Krasnow RE, Swan GE, Reed T (2015). Higher usual alcohol consumption was associated with a lower 41-y mortality risk from coronary artery disease in men independent of genetic and common environmental factors: the prospective NHLBI Twin Study. Am. J. Clin. Nutr..

[52] Jaffe AE, Irizarry RA (2014). Accounting for cellular heterogeneity is critical in epigenome-wide association studies. Genome Biol..

[53] D'Agostino RB (2008). General cardiovascular risk profile for use in primary care: the Framingham Heart Study. Circulation.

[54] Shtatland, E. S. & Barton, M. B. Information as a Unifying Measure of Fit in SAS^®^ Statistical Modeling Procedures. *Northeast SAS Users Group NESUG’97 Proceedings*, 875–880 (1997). https://www.lexjansen.com/nesug/nesug97/stat/shtatlan.pdf. (Accessed 16 Feb 2022)

[55] Efron B, Tibshirani R (1986). Bootstrap methods for standard errors, confidence intervals, and other measures of statistical accuracy. Stat. Sci..

[56] Aryee MJ (2014). Minfi: A flexible and comprehensive Bioconductor package for the analysis of Infinium DNA methylation microarrays. Bioinformatics.

