# Supplementary material for: Methylation at CpG sites related to growth differentiation factor-15 was not prospectively associated with cardiovascular death in discordant monozygotic twins
Source: Sci Rep. 2022 Mar 15;12:4410. doi: 10.1038/s41598-022-08369-9 (PMC8924170; doi:10.1038/s41598-022-08369-9)
Supplement: Supplementary file 1 — Supplementary Information. [file 41598_2022_8369_MOESM1_ESM.docx]

| Table S1. Hazard ratio (HR) per 10% unit increase in methylation beta value derived from the regression coefficient from conditional logistic model among monozygotic twins discordant for cardiovascular and coronary heart deaths in the original sample. *HR* hazard ratio. Adjusted hazard ratios were obtained after controlling for a principal component score representing age, years of education, smoking status, body mass index, systolic blood pressure, low-density lipoprotein cholesterol, high-density lipoprotein cholesterol, use of antihypertensives, presence of diabetes, and leukocyte subtype composition. | | | |
| --- | --- | --- | --- |
|  | **HR (95% Confidence Interval)** | **p value**  **for CpG site** | **p value for the joint effect of predictors** |
| stringently defined monozygotic twin pairs discordant for total cardiovascular death | | | |
| cg13033858 |  |  |  |
| Crude | 0.90 (0.17, 4.82) | 0.90 | 0.90 |
| Adjusted | 1.98 (0.20, 19.78) | 0.56 | 0.47 |
| cg16936953 |  |  |  |
| Crude | 1.60 (0.30, 8.60) | 0.58 | 0.58 |
| Adjusted | 9.60 (0.35, 266) | 0.18 | 0.15 |
| cg17150809 |  |  |  |
| Crude | 1.14 (0.10, 13.26) | 0.91 | 0.91 |
| Adjusted | 1.73 (0.12, 24.46) | 0.69 | 0.52 |
| cg18608055 |  |  |  |
| Crude | 0.57 (0.09, 3.50) | 0.54 | 0.53 |
| Adjusted | 1.10 (0.10, 12.07) | 0.94 | 0.56 |
| primarily defined monozygotic twin pairs discordant for coronary heart death | | | |
| cg13033858 |  |  |  |
| Crude | 0.44 (0.08, 2.31) | 0.33 | 0.30 |
| Adjusted | 0.50 (0.07, 3.30) | 0.47 | 0.36 |
| cg16936953 |  |  |  |
| Crude | 1.21 (0.35, 4.17) | 0.76 | 0.76 |
| Adjusted | 2.03 (0.41, 9.94) | 0.38 | 0.31 |
| cg17150809 |  |  |  |
| Crude | 1.37 (0.22, 8.54) | 0.74 | 0.73 |
| Adjusted | 1.86 (0.25, 13.4) | 0.54 | 0.40 |
| cg18608055 |  |  |  |
| Crude | 0.51 (0.10, 2.63) | 0.42 | 0.40 |
| Adjusted | 0.82 (0.12, 5.73) | 0.84 | 0.48 |

| stringently defined monozygotic twin pairs discordant for coronary heart death | | | | |
| --- | --- | --- | --- | --- |
| cg13033858 |  |  | |  |
| Crude | 0.63 (0.12, 3.33) | 0.58 | 0.57 | |
| Adjusted | 0.91 (0.12, 6.99) | 0.93 | 0.42 | |
| cg16936953 |  |  |  | |
| Crude | 0.52 (0.08, 3.31) | 0.49 | 0.47 | |
| Adjusted | 0.89 (0.11, 6.96) | 0.91 | 0.42 | |
| cg17150809 |  |  |  | |
| Crude | 0.21 (0.01, 6.07) | 0.37 | 0.35 | |
| Adjusted | 0.30 (0.01, 9.41) | 0.50 | 0.33 | |
| cg18608055 |  |  |  | |
| Crude | 0.32 (0.05, 2.22) | 0.25 | 0.21 | |
| Adjusted | 0.49 (0.06, 4.26) | 0.52 | 0.34 | |

| **Table S2. The Akaike information criterion (AIC) for the model selection based on the first three principal components for the conditional logistic model among monozygotic twins discordant for total cardiovascular deaths in the original sample.**  *PC* principal component. The *PC1*, *PC2*, and *PC3* represent the first three principal components. *PC1* the first *PC*, *PC2* the second *PC*; *PC3* the third *PC*. The model selection based on the AIC value reflects the trade-off between the goodness of fit and the complexity of the model. *The smallest AIC, the most parsimonious model. | | | | |
| --- | --- | --- | --- | --- |
| **CpG sites** | **Predictors in the Model** | | | |
|  | **CpG** | **CpG+PC1** | **CpG+PC1+PC2** | **CpG+PC1+PC2+PC3** |
| cg13033858 | 27.820 | 27.801* | 29.796 | 31.781 |
| cg16936953 | 26.563 | 23.681* | 25.407 | 26.341 |
| cg17150809 | 27.952 | 26.043* | 27.956 | 27.704 |
| cg18608055 | 28.328 | 26.889* | 28.824 | 30.642 |
|  | | | | |

**Figure S1. Within-pair differences (i.e., pair-wise differences) in age at death for 19 monozygotic twin pairs discordant for death from total cardiovascular diseases**
